# Are *Babesia *a risk factor for blood products in an alpine area?

**DOI:** 10.1186/1756-3305-7-S1-O37

**Published:** 2014-04-01

**Authors:** ST Sonnleitner, R Baumgartner, R Edelhofer, H Schennach, M Bednarska, K Pister, G Walder

**Affiliations:** 1 Dr. Gernot Walder GmbH, Außervillgraten, Austria; 2 Institute of Parasitology, University of Veterinary Medicine, Vienna, Austria; 3 Central Institute for Blood Transfusion and Immunology, Innsbruck Medical University, Innsbruck, Austria; 4 Department of Parasitology, University of Warsaw, Warsaw, Poland; 5 Comparative Tropical Medicine and Parasitology, University of Munich, Munich, Germany; 6 Department of Hygiene, Medical Microbiology and Social Medicine, Innsbruck Medical University, Innsbruck, Austria

## 

After malaria, babesiosis is the second most common transfusion-transmitted vector-borne disease. This study investigates seroprevalence rates to *Babesia divergens *and *B. microti *in the Tyrol and assesses the risk of blood products being contaminated by either agent.

The area of investigation comprises the Austrian part of Tyrol. A number of 988 sera were tested for IgG antibodies against *B. divergens *and *B. microti *by in-house immunofluorescence assays (IFA). IFA-slides were tested by using commercially available hyperimmunesera.

Collection of questing ticks was performed in summer 2009 by about 120 volunteers among hunters at 25 sampling sites over a period of three months by flagging.

Of 988 sera, 21 (2.1%) were positive in IFA against the *B. divergens*-complex at titres of 1:128 or higher and 5 (0.5%) were positive in IFA against *B. microti*.

Under the presumption of a long-lasting immune response we can expect 0.5 (±0.2, 95%) seroconversions against *B. divergens *per 10.000 persons per year. For *B. microti *the same calculation results in 0.1 (±0.08, 95%) seroconversions per 10.000 persons per year. B. divergens The risk of a blood donation being contaminated by *B. divergens *or *B. microti *is estimated to be 24.2 and 5.8 per 100,000 blood donations.

The present study shows that the local population comes into seroreactive contact with at least one member of the *B. divergens*-complex and - to a lesser extent - *B. microti*. To our knowledge, it is the first demonstration of *B. venatorum *in the Tyrols. Thus, and as vector-borne diseases are subjected to dynamic changes, we recommend re-assessment of the risk of transfusion-mediated infections on a regular basis and to introduce PRT for blood components like platelets.

